# Categorical judgments do not modify sensory representations in working memory

**DOI:** 10.1371/journal.pcbi.1008968

**Published:** 2021-06-01

**Authors:** Long Luu, Alan A. Stocker

**Affiliations:** 1 Zuckerman Institute, Columbia University, New York, United States of America; 2 Department of Psychology, University of Pennsylvania, Philadelphia, United States of America; Stockholm University, SWEDEN

## Abstract

Categorical judgments can systematically bias the perceptual interpretation of stimulus features. However, it remained unclear whether categorical judgments directly modify working memory representations or, alternatively, generate these biases via an inference process down-stream from working memory. To address this question we ran two novel psychophysical experiments in which human subjects had to reverse their categorical judgments about a stimulus feature, if incorrect, before providing an estimate of the feature. If categorical judgments indeed directly altered sensory representations in working memory, subjects’ estimates should reflect some aspects of their initial (incorrect) categorical judgment in those trials. We found no traces of the initial categorical judgment. Rather, subjects seemed to be able to flexibly switch their categorical judgment if needed and use the correct corresponding categorical prior to properly perform feature inference. A cross-validated model comparison also revealed that feedback may lead to selective memory recall such that only memory samples that are consistent with the categorical judgment are accepted for the inference process. Our results suggest that categorical judgments do not modify sensory information in working memory but rather act as top-down expectations in the subsequent sensory recall and inference process.

## Author summary

Humans have the tendency to remain consistent with their previous categorical judgments when recalling memorized evidence. This tendency gives rise to many known forms of consistency and confirmation biases. Using a detailed computational model comparison, we show that these biases do not reflect distortions in the memory representation of the evidence but rather result from a biased interpretation of the evidence during memory recall. Our results are important as they suggest that consistency biases are malleable, even reversible, which has implications for our understanding of economic and cognitive decision-making processes and even society at large.

## Introduction

Human visual perception is biased by the statistical regularities in the sensory input. Many studies have demonstrated that these biases can be well accounted for by assuming that perception is an inference process that optimally trades-off sensory uncertainty with prior expectations reflecting these statistical regularities [1–6]. However, perception is not only biased by more objective, statistical prior expectations but also by the observer’s subjective categorical assessment of a visual feature. For example, in a task sequence where subjects first had to make a categorical judgment about a stimulus feature before providing an estimate of the feature value (motion direction [7–9], orientation [10, 11] or numerosity [9, 12, 13]), the resulting estimates showed systematic biases in favor of their preceding category choices. Similar bias patterns have also been found in sequential tasks that did not explicitly involve categorical judgments [8, 14–17] or estimates [18, 19], suggesting that they are a wide-spread and general phenomenon in hierarchical perceptual inference [20]. A general theory proposes that these bias patterns emerge because inference is intrinsically a top-down process where low-level feature estimates are conditioned on the observer’s preceding, (explicit or implicit) high-level categorical judgment [10, 16, 20–22]. Embodying this theory, a “self-consistent” Bayesian observer model [21] has performed remarkably well in quantitatively capturing the rich and diverse perceptual behavior of individual human subjects in these tasks [10]. The model assumes that a categorical judgment acts as a subjective prior that conditions the inference process of the stimulus feature.

The motivation for humans to employ such a conditioned inference strategy is unclear, in particular because it is sub-optimal with regard to estimation accuracy. However, common to all tasks described above is that they are sequential processes that operate over time and require a working memory representation of the stimulus. Top-down conditioning may help to restore working memory that is corrupted by noise [10, 16] although a detailed analysis revealed that this benefit does not always lead to improved accuracy [20]. One established advantage of conditioning is that the observer’s interpretation of the stimulus remains self-consistent across all levels of representation (e.g., from category to feature level) despite corruption by memory or late noise [10, 20]. Conditioning may thus reflect a general inference strategy of the mind to avoid dissonant interpretations of the world at any moment in time [23, 24].

With the current work, we aimed at identifying how and at what level a categorical judgment interacts with working memory representations in a way that is consistent with the observed post-decision biases. Specifically, we tested whether a categorical judgment directly modifies sensory representations in working memory or not. We used an experimental design similar to our previous study [10, 22]: After presentation of an orientation stimulus, subjects first made a categorical judgment about the overall stimulus orientation and then subsequently provided an estimate of the orientation. In contrast to our previous work, however, subjects were given feedback about their categorical judgment in each trial. If categorical judgments modified working memory representation, we would expect subjects’ estimates to reflect these modifications in trials in which they had to change their categorical judgment because their initial assessment was incorrect. Our experimental results did not show any evidence that a categorical judgment updates and modifies working memory representations. Rather, subjects were able to flexibly change their categorical choice (if incorrect) and recombine it with stimulus information stored in working memory as predicted by the self-consistent observer model [10, 21]. This suggests that categorical and feature information have rather independent neural representations in working memory, which can be flexibly recombined in the perceptual inference process if necessary.

## Results

**Fig 1:**
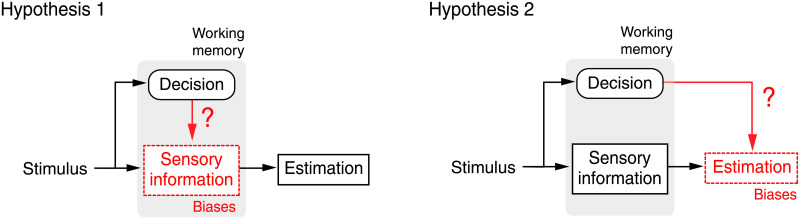
Origins of post-decision estimation biases. Are choice-induced estimation biases the result of a modified sensory representation in working memory (Hypothesis 1) or rather of a computational inference process downstream (Hypothesis 2)? We run psychophysical experiments in which subjects had to reverse their categorical decision if incorrect according to feedback. Under Hypothesis 1, traces of the first (incorrect) decision should be visible in subjects’ estimates.

Fig 1 outlines the two complementary hypotheses. As the observer performs an implicit or explicit high-level categorical judgment, sensory information about the stimulus value is stored in working memory and then subsequently used to also perform an estimate of the stimulus feature. This estimate is typically biased by the preceding categorical decision [7, 8, 10]. We previously demonstrated that at the computational level, a self-consistent observer model can accurately account for human subjects’ behavior both in the decision (if explicit) and the estimation task [10]. The model assumes that the observer’s categorical judgment acts as a subjective prior, which conditions feature inference to be consistent with the categorical judgment. However, at the process level, it remained unclear whether stimulus information and the categorical decision are combined in a non-separable representation in working memory (Hypothesis 1), or whether the two are separately represented and maintained, and then independently combined down-stream of working memory (Hypothesis 2).

To test the hypotheses, we performed two psychophysical experiments similar in design to our previous experiments [10]. In both experiments, subjects were briefly presented with a noisy visual orientation stimulus. They then first had to decide whether the stimulus’ orientation was clockwise (cw) or counter-clockwise (ccw) of a reference orientation before providing an estimate of the orientation by adjusting a cursor. Different to our previous experiments, however, subjects received valid feedback on their categorical judgment before they performed the estimate. Note, that feedback was delivered with a delay of 500 ms after subjects’ category choice, thus providing time for the decision to potentially update sensory information in working memory. Subjects were instructed to consider the feedback when providing their estimates, which they always did. Both experiments included stimuli with different levels of stimulus uncertainty, and only differed in that in Experiment 1 the range of stimulus orientations was symmetric around the reference whereas it was asymmetric in Experiment 2.

### Predictions

Both hypotheses make identical predictions for trials where subjects’ categorical judgment was correct. Given previous results, we expected subjects’ behavior to be well described with the self-consistent observer model [10, 21]. However, predictions differ for trials where that judgment was incorrect and subjects had to change their categorical assessment.

**Fig 2:**
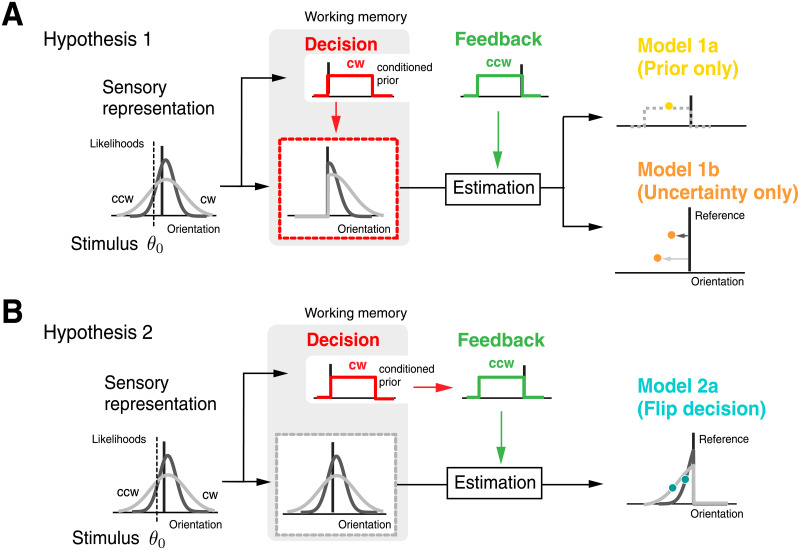
Modeling stimulus estimation after receiving negative feedback. (A) Hypothesis 1: After making a categorical judgment, working memory is updated to only reflect the stimulus information consistent with the decision (i.e., the posterior). We considered two models of how an observer forms an estimate under this condition upon receiving feedback that the decision was incorrect. Model 1a (prior only) assumes that the observer has access to the stimulus prior associated with the correct category. In contrast, Model 1b (uncertainty only) assumes that the observer only has knowledge of the overall sensory uncertainty. As a crude approximation of behavior in correct trials for which larger stimulus noise generally results in larger repulsive biases, we made the heuristic assumption that the observer picks an estimate away from the reference orientation that corresponds to this uncertainty value. (B) Hypothesis 2: Sensory representation (i.e. the entire likelihood function) is preserved in memory and only the conditioned prior is updated when feedback indicates an incorrect choice. Model 2a (flip decision) posits that the observer can freely recombine the recalled sensory representation with the updated prior to perform a Bayesian estimate.

Hypothesis 1 assumes that sensory information about stimulus orientation and the categorical judgment are *jointly and inseparably* represented in working memory. Specifically, we make the assumption that the sensory information in working memory is updated after the decision to reflect the trial posterior distribution (Fig 2A). According to the self-consistent observer model the posterior is zero for all stimulus orientations that are not in agreement with the categorical judgment. Thus, when receiving feedback indicating that the categorical choice was incorrect, the observer is tasked to generate an estimate of the stimulus orientation without having any specific information about the true stimulus orientation on the correct side. We considered two models for how the observer may perform an estimate under this condition, each reflecting the separate use of the two sources of stimulus information available to a Bayesian observer—stimulus prior and sensory uncertainty. Model 1a assumes that the observer has access to their (subjective) prior distribution over stimulus orientations corresponding to the correct stimulus category and selects an orientation estimate that represents the mean of this prior, which corresponds to an optimal estimate under mean squared-error loss based on prior information only. The predicted estimates are, on average, identical across all stimulus orientations and independent of stimulus noise, and their variance is only determined by the subject’s motor noise. Note that we did not consider the possibility that an observer would simply pick a random orientation estimate on the correct side of the reference because the data clearly refutes such a trivial model. In contrast, Model 1b assumes that although the observer has no longer access to specific information about stimulus orientation on the correct side of the reference, it still has a sense of the overall level of sensory uncertainty in a trial. Specifically, we assume that the observer picks an estimate relative to the reference orientation according to sensory uncertainty (i.e., the standard deviation *σ*_*s*_ of the assumed Gaussian noise distribution—see Methods). This heuristic constitutes a crude approximation of subjects’ behavior in correct trials where larger stimulus noise results in larger biases away from the reference orientation. The predicted estimates depend on sensory uncertainty but do not reflect any other aspect of the stimulus.

Hypothesis 2 assumes that sensory information and the categorical decision are independently represented in working memory (Fig 2B). We considered a model (Model 2a) where, upon receiving negative feedback, the observer can flexibly recombine the sensory information stored in working memory (i.e. the likelihood function) with the correct category prior in order to estimate stimulus orientation. This model is equivalent to the self-consistent observer model under the assumption that the observer has the flexibility to simply flip the categorical choice. As a result, nothing in the inference process is affected by the observer’s initial, incorrect categorical judgment. Later we will introduce and test two variants of this model (Model 2b and 2c).

We evaluated the two general hypotheses based on a particular strong form of cross-validation: We fit only the data representing correct trials (i.e., trials in which subjects provided a correct categorical judgment) and then used the fit parameter values to predict estimation behavior in incorrect trials for all above models. Models are fully constrained by the parameters of the self-consistent observer model, thus the model comparison is parameter-free.

### Experiment 1: Results and model analysis

**Fig 3:**
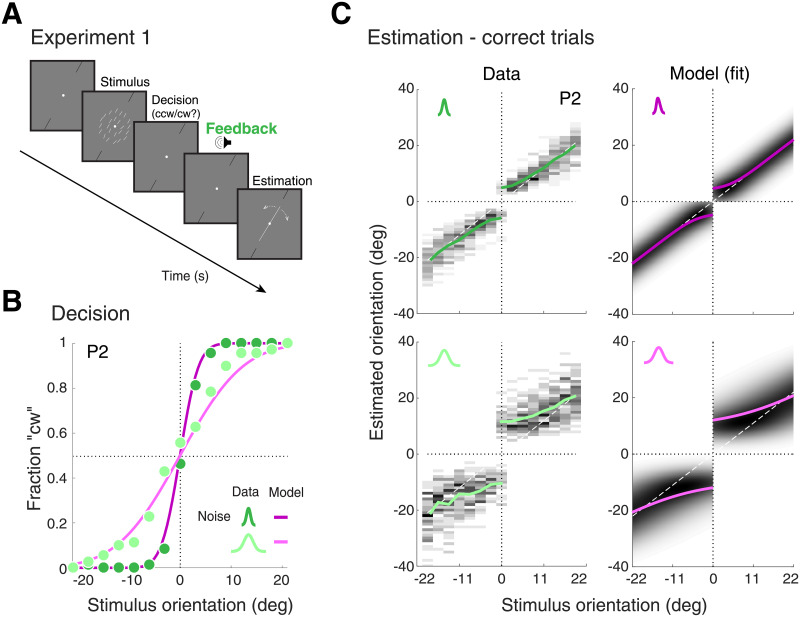
Experiment 1—Data and model fits for correct trials. (A) The stimulus consisted of an array of line segments whose orientations were sampled from a Gaussian centered at the stimulus orientation *θ*. Stimuli had one of two levels of noise corresponding to different widths of the Gaussian (*σ*_*s*_ ≔ {3, 18} deg). Stimulus orientation was uniformly distributed within a range ± 21 deg around the reference orientation. Subjects first reported whether the stimulus orientation was cw/ccw of the reference orientation (indicated by two black lines). 500 ms after reporting their decision, they received auditory feedback indicating whether their decision was correct or not. Instructed to take feedback into account, subjects then provided an estimate of stimulus orientation by adjusting a line cursor. (B) Categorical judgment data (green circles) and the self-consistent observer model fit (purple lines) for typical subject P2. Darker shades represent data/fit for lower stimulus noise. As expected, the slope of the psychometric function is steeper for a lower noise level. Stimulus orientation is relative to the reference orientation. (C) Estimate distributions and the predicted distributions of the fit model for typical subject P2 (colored lines represent mean estimates). Subjects show the characteristic repulsive bias away from the reference, confirming the results of previous studies [7, 8, 10]. Note that the model was jointly fit to the decision and estimation data. See S1 Fig for data and model fits of all other subjects.

Five subjects (P1–5; P1 non-naïve) participated in Experiment 1. In each trial, an array of small, oriented line segments was briefly (500 ms) presented (Fig 3A). After the stimulus disappeared, subjects had to indicate whether stimulus orientation was cw/ccw relative to a displayed reference orientation. Auditory feedback indicated whether the categorical judgment was correct or not. Subjects were instructed that feedback was always valid, which it was. After receiving feedback, subjects had to report perceived stimulus orientation by adjusting a joystick.

Fig 3B and 3C show the categorical judgment and estimation data for a typical subject (P2). Estimate distributions (Fig 3C) only reflect correct trials. Data confirm previous results showing estimates that are biased away from the reference orientation with larger biases for higher stimulus uncertainty and stimulus values closer to the reference [7, 8, 10]. Note that the characteristic bimodal estimate distributions are a clear indication that these biases are real and not an artifact caused by separately analyzing estimates associated with each categorical judgment. Shown are also the predictions of the best fitting self-consistent observer model. Confirming our previous findings, the model well explains both the psychometric function of the categorical judgment as well as the bimodal distributions of orientation estimates [10]. Data and individual model fits for all other subjects are shown in S1 Fig.

**Fig 4:**
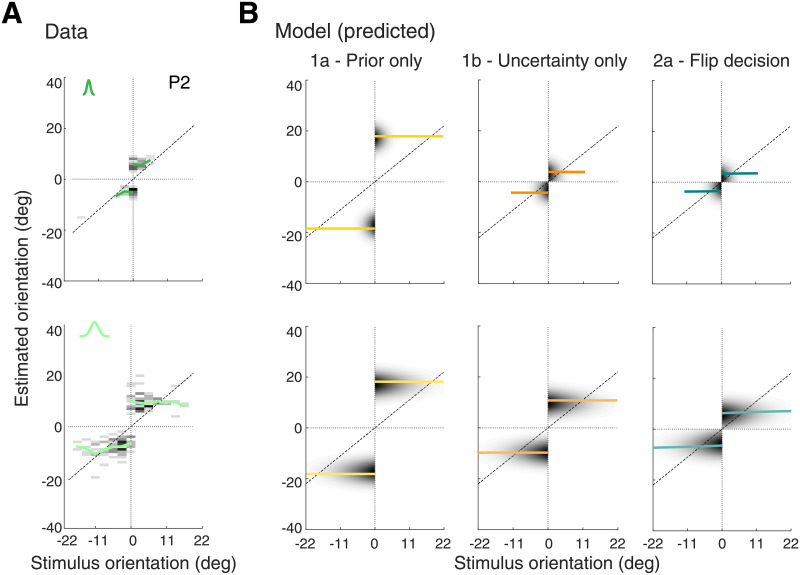
Experiment 1—Data and model predictions for incorrect trials. (A) Estimate distributions for incorrect trials (subject P2). Top row corresponds to low, bottom row to high stimulus noise. For stimulus orientations further away from the reference, the data get sparser because subjects made less incorrect decisions. Mean estimates (green lines; computed as the mean across all trials at each tested stimulus orientation) are approximately constant on each side of the decision boundary with magnitude and variance larger for higher stimulus noise. Also, distributions are long-tailed away from the reference orientation (see S8 Fig). (B) All models predict estimate distributions with approximately constant mean and variance across stimulus orientation. For Model 1a (prior only), however, mean and variance are independent of stimulus uncertainty with the variance determined only by motor noise. In contrast, the remaining two models (1b and 2a) correctly predict larger estimation means for higher stimulus noise, although only Model 2a predicts long-tailed distributions. Both models provide a good account of the data, which demonstrates that subjects indeed have access to information about sensory uncertainty after receiving negative feedback and can incorporate this information in their estimates. See S2 Fig for data of all other subjects.

**Fig 5:**
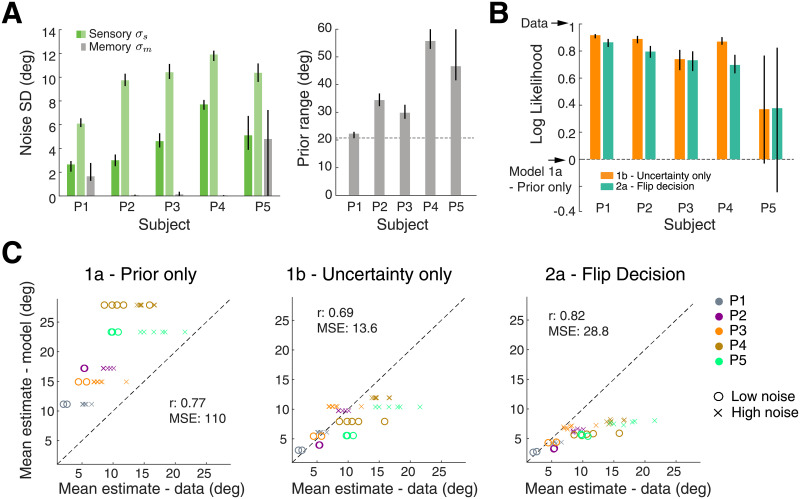
Experiment 1—Fit model parameters and prediction accuracy for individual subjects. (A) Fit noise levels and prior widths. While there is substantial inter-subject variability, the range of parameters values is consistent with our previous study [10]. (B) Log-likelihood comparison between the models. Values are normalized relative to the log-likelihood values of Model 1a (prior only) and the ‘omniscient’ model (‘Data’—i.e. the likelihood value based on the empirical probabilities; see Methods for details). Model 1a performs substantially worse than the other models for all subjects. However, log-likelihoods for Models 1b and 2a are not statistically different except for subject P4. All errorbars in (A) and (B) indicate 95% confidence intervals computed over 200 bootstrapped samples. (C) Scatter plots comparing the mean estimates between models and data. Each point corresponds to one experimental condition (stimulus noise and orientation) of one subject. Although all models can approximately explain the trend in data, the predictions of Model 1a are substantially off. Compared to Model 1b, Model 2a has a higher correlation but is worse when comparing mean-squared error (MSE) between model and data mean. Model 2a (flip decision) tends to underestimate the magnitude of subjects’ estimates proportional to stimulus magnitude.

Next, we predicted the estimate distributions for incorrect trials for all models based on the fit model parameters. Fig 4 shows the experimental data for typical subject P2 (see S2 Fig for data of all other subjects) together with the model predictions (parameters shown in Fig 5A). The data reveal a few noteworthy characteristics. First, subjects’ orientation estimates were on average fairly independent of stimulus orientation, yet clearly different for the two stimulus noise conditions. Second, the distributions for any given stimulus orientation are long-tailed away from the reference. Naively, one might interpret the relative constant mean of the estimates as an indication that subjects did not or could not reuse sensory information in incorrect trials, and thus simply pointed the cursor somewhere to the side of the reference signaled by feedback. However, the fact that magnitude and variance of the estimates were substantially larger in the high stimulus noise condition is inconsistent with such interpretation. Because the stimulus noise conditions were randomly interleaved, it rather demonstrates that the computation of subjects’ estimates incorporates sensory uncertainty on a per-trial basis [10]. A more detailed comparison across subjects further shows that subjects’ estimates are strongly correlated with their individual prior widths and levels of sensory uncertainty extracted from the model fit to their correct trial data (S5 Fig). This suggests that also in incorrect trials, subjects’ estimates reflect the result of an inference process that combines both sensory information retained in working memory and learned prior expectations.

All models predict average estimates with no (Model 1a and 1b) or very weak (Model 2a) dependence on stimulus orientation, thus matching a key characteristic of the data (Fig 4). While stimulus independent estimates are a direct consequence of the main assumption of Hypothesis 1, it might not be intuitive why Hypothesis 2 makes a similar prediction. After all, the hypothesis assumes that the observer maintains a full sensory representation of the stimulus and thus has as much sensory information at hand as in correct trials. The very weak dependence is explained by the fact that the Bayesian estimates are computed with the tail of the likelihood function because in incorrect trials the likelihood mean is on the incorrect side (hence the incorrect categorical decision). Because the mean of the tail only very weakly depends on the likelihood mean (that on average is equal to the stimulus orientation), the posterior mean and thus the estimate also only weakly depends on the likelihood mean. Note, that the limited amount of incorrect trial data at larger stimulus orientations does not allow us to state with significance whether such weak dependence is present in the data or not. Predictions of Model 1a (prior only) markedly differ from the data in that the predicted estimate distributions are identical for both stimulus noise levels (Fig 4B); the means are identical with the mean of subjects’ individual prior distributions (Fig 5A). In contrast, Model 1b (uncertainty only) and 2a (flip decision) both correctly predict larger estimate magnitudes for high stimulus noise conditions.

We extended the above qualitative model comparison with a quantitative analysis across all subjects. We fit correct trial data for each individual subject and then used the fit model parameters to predict estimate distributions for the incorrect trials. Fig 5A shows the fit model parameters for all subjects. The parameter values are consistent with those in our previous study [10] providing further confirmation of the self-consistent observer model. Because all models can predict the full distribution of estimates (i.e. they are observer models), we then computed the likelihood of each model’s prediction given the incorrect trial data. Fig 5B shows these model likelihoods for each subject normalized to the range set by the likelihoods for Model 1a and an omniscient model (‘Data’). The latter can be thought of as the data explaining themselves, that is, a ‘model’ defined by the empirical probabilities of the data. It provides a natural upper bound for model performance (see Methods). The quantitative evaluation confirms our initial, qualitative comparison. Model 1a (prior only) is significantly worse than the other models. Although Model 1b (uncertainty only) performs best for most subjects, it is not statistically different from Model 2a (flip decision) except for subject P4.

We also analyzed which model better captures the overall bias patterns in the data by comparing the mean estimates for each stimulus condition (stimulus noise and stimulus orientation) with the corresponding predictions of the models. Fig 5C shows the comparison for all subjects including the correlation and mean squared-error (MSE) between model predictions and data. Although correlations are high for all models, Model 1a performs rather poorly compared to the other two models. Model 2a (flip decision) performs best in terms of correlation but not MSE, showing consistent and proportional underestimation of subjects’ stimulus estimates.

In summary, both the experimental and analytical results of Experiment 1 show that, at minimum, information about trial-by-trial sensory uncertainty is retained in subjects’ working memory. Furthermore, they suggest that this information must be stored in a way that is independent of a particular (high-level) categorical interpretation of the stimulus and thus can be flexibly recombined with the feedback information. However, the data are approximately equally well explained by Model 1b (uncertainty only) and Model 2a (flip decision). Thus it remains unclear whether the estimates only reflect the observer’s knowledge of sensory uncertainty or, alternatively, are the result of an inference process that combines information about sensory uncertainty with prior information. Which is why we conducted Experiment 2.

### Experiment 2: Results and model analysis

Experiment 2 was similar to Experiment 1 except that the range of stimulus orientations relative to the reference orientation was shifted to the cw side ([-12, 30] deg). Note that the stimulus distribution was still uniform on either side and the fraction of stimulus orientations being cw or ccw was still 0.5. If subjects were able to flexibly apply knowledge of this stimulus range asymmetry then their estimates should also reflect this asymmetry both in correct *and* incorrect trials.

**Fig 6:**
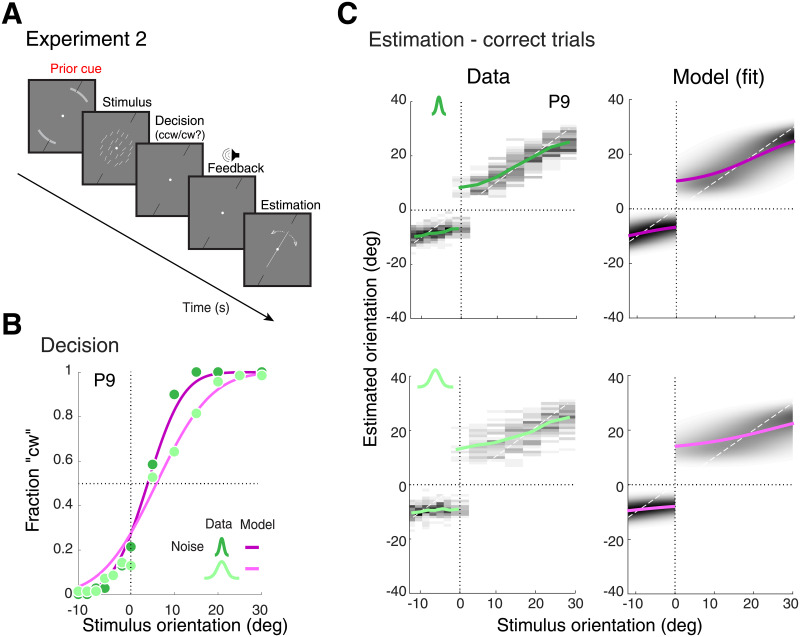
Experiment 2—Data and model fits for correct trials. (A) Experiment 2 was identical to Experiment 1 except that the stimulus range was asymmetric around the reference ([-12, 30] deg, positive values indicating cw orientations relative to the reference). Subjects were reminded about the asymmetry at the beginning of each trial by explicitly showing the actual stimulus range (gray arcs). Stimulus noise levels corresponded to two different widths of the stimulus array distribution (*σ*_*s*_ ≔ {6, 18} deg). (B) Psychometric functions representing subjects’ categorical choice behavior (typical subject P9). Green circles indicate the data and purple lines indicate the fit with the self-consistent observer model. Darker colors represent conditions with lower stimulus noise. Note that the subjects’ decision probability of choosing cw is smaller than 0.5 for stimuli at the reference orientation. This matches the prediction of an optimal decision-maker given the asymmetric stimulus range even with equal prior probability for each category. When fitting, we allowed the category prior probability to be a free model parameter although fit values were close to 0.5 (see Fig 7A). (C) Measured estimate distributions and the predictions of the fit self-consistent model for correct trials only (colored lines: mean). Estimates on the ccw side are closer to the reference and less repulsed. This is well captured by the model and thus can be explained as the effect of the asymmetric prior in the inference process. See S3 Fig for data and model fits of all other subjects.

Fig 6A outlines the structure of a single trial in Experiment 2. Subjects were trained prior to the actual experiment in order to learn the asymmetric stimulus distribution. In addition, subjects were reminded of the actual stimulus range with a visual cue at the beginning of each trial [10]. The rest of the trial design was identical to Experiment 1. Seven subjects participated in the experiment of which one (non-naïve subject P1) also participated in Experiment 1.

Fig 6B shows a subject’s categorical choice behavior and the fit with the self-consistent observer model (typical subject P9; see S3 Fig for data and fits of all other subjects). The closer the stimulus orientation is to the reference and the larger the stimulus uncertainty, the more frequently incorrect choices occurred. Fig 6C shows the estimate distributions (correct trials only) for each stimulus noise level together with the predictions of the self-consistent observer model. Estimates are overall larger for stimulus orientations on the cw side of the reference. This is particularly visible in the high noise condition and indicates that subjects took the asymmetric stimulus range into consideration when estimating stimulus orientation. The model well captures this characteristic suggesting that the asymmetry in estimation bias is fully explained by a Bayesian inference process with corresponding asymmetric prior distributions. Fit prior parameters confirm this, showing values that are close to the true orientation range (Fig 8A). The results provide additional confirmation of the self-consistent observer model as it has not been tested before for asymmetric stimulus priors.

**Fig 7:**
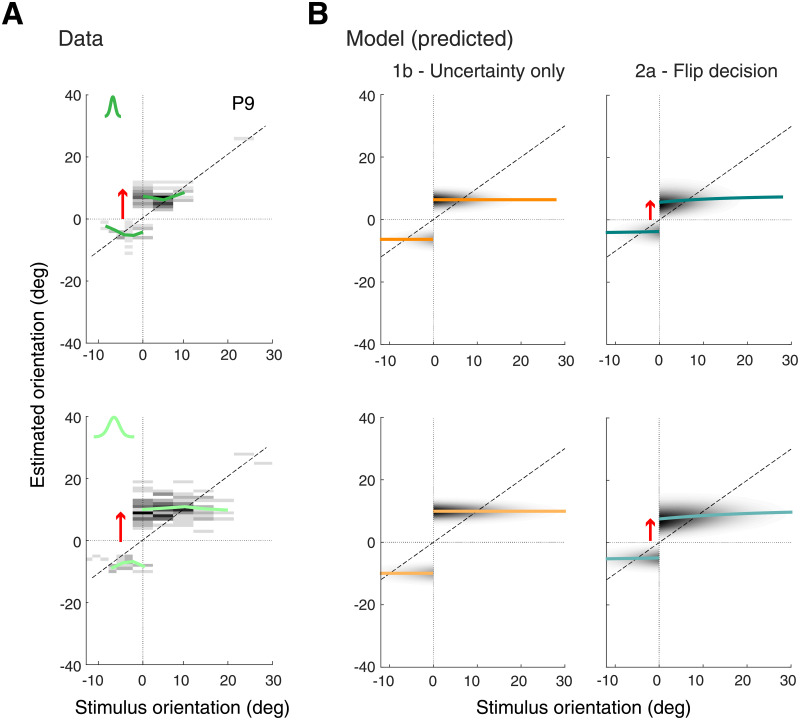
Experiment 2—Data and model predictions for incorrect trials. (A) Estimate distributions for incorrect trials (typical subject P9; see S4 Fig for data of all other subjects). As in Experiment 1, estimates are approximately constant and independent of stimulus orientation. However, the distributions show a clear asymmetry with estimates on the cw side being further away from the reference orientation than on the ccw side (red arrow). This pattern suggests that subjects were able to flexibly change and incorporate the correct prior in their estimate according to feedback. (B) Model 2a correctly predicts this asymmetry while Model 1b does not. Red arrows indicates the side with larger repulsive biases. Solid colored lines reflect mean estimates.

For incorrect trials, observing the same asymmetry as in correct trials with larger estimates on the cw side would imply that subjects can flexibly switch their categorical prior to the one corresponding with feedback when inferring stimulus orientation. This is indeed what we found (Fig 7A): subjects’ estimates in incorrect trials show the same asymmetry as in the correct trials; the average estimate is larger on the cw than on the ccw side of the reference (red arrow). Models 1b and 2a, however, make markedly different predictions (Fig 7B). While Model 2a (flip decision) follows the data, Model 1b (uncertainty only) predicts no asymmetry as it does not take prior information into account.

**Fig 8:**
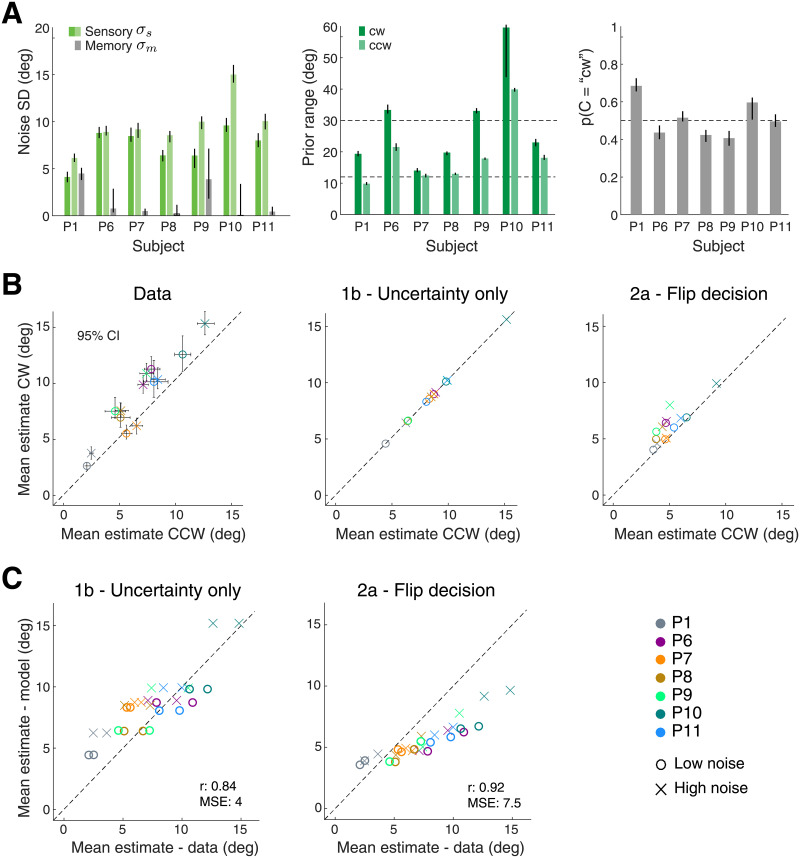
Experiment 2—Fit model parameters and prediction accuracy for individual subjects. (A) Sensory (*σ*_*s*_) and memory (*σ*_*m*_) noise parameters of individual subjects (correct trials only). The prior width is consistently higher on the cw side for all subjects. In contrast to Exp. 1, the category prior *p*(*C* = “cw”) was a free parameter. On average values are close to the statistically correct value of 0.5. (B) Mean estimates (averaged across stimulus orientations) in incorrect trials are significantly larger on the cw side for all except one subject (P7). Errorbars reflect the 95% bootstrapped sample interval. Model 2a correctly predicts this pattern but Model 1b does not (no prior). (C) Model 1b performs better in terms of MSE between data and model mean estimates but Model 2a’s predictions show a higher correlation. However, the inability to account for the asymmetry in the estimate data is sufficient evidence to rule out Model 1b. Predictions of Model 2a show the same tendency to underestimate estimation magnitudes as in Exp. 1.

We also performed a quantitative model analysis for data of individual subjects. The fit model parameters are presented in Fig 8A. Noise parameters are consistently larger for the high-noise stimulus. Fit prior parameters suggest that the behavior of all subjects reflects, to various degrees, knowledge of the asymmetric stimulus range and, on average, the correct category prior (0.5). Fig 8B shows subjects’ mean estimates in incorrect trials and the corresponding model predictions. Like subject P9, the other subjects (5 out of 6) also show mean estimates that are significantly larger on the cw compared to the ccw side. The predictions of Model 2a well capture this pattern due to the asymmetric prior in the inference process. Model 1b, however, does not. This suffices to rule out Model 1b as an accurate account of subjects’ behavior even though overall prediction accuracy (e.g. MSE and correlation between data and model mean estimates Fig 8C) are matched or even slightly better compared to those of Model 2a. As in Experiment 1, subjects’ estimates are strongly correlated with their individual prior widths and levels of sensory uncertainty extracted from the model fit to their correct trial data (S6 Fig). This is further evidence that subjects’ estimates in incorrect trials are the result of an inference process that combines sensory information retained in working memory with learned prior expectations.

In summary, the results of Experiment 2 suggest that subjects were able to combine the initial sensory representation and its uncertainty with the new, correct category assignment in an inference process that considers both stimulus uncertainty and prior. It extends previous results [25] by demonstrating that stimulus uncertainty and priors are not only represented in working memory but are represented independently and in a form that allows subjects to **flexibly switch and recombine** them to perform perceptual inference. This is an important finding that has implications for understanding working memory function beyond the current experimental task.

### Resampling or reweighting of sensory evidence due to negative feedback

Model 2a well predicts the key characteristics of the behavioral data, although quantitatively the predictions slightly but consistently deviate from subjects’ estimates in both experiments. The predicted estimates are generally smaller than subjects’ estimates by an amount that is approximately proportional to the estimation magnitudes (see Figs 5C and 8C). The high correlation between the predictions and the data (R = 0.82 and 0.92, respectively) indicates that the fit model parameters extracted from correct trials are in excellent agreement with subjects’ behavior in incorrect trials, therefore the systematic deviations are likely caused by an additional computational process that is activated whenever subjects receive negative feedback. The notion that negative feedback causes more than a change of the conditioned prior is further supported by a comparison of the subjects’ response times (RT) between correct and incorrect trials (S10 Fig); when receiving negative feedback, subjects RTs were on average 0.5 s longer. In the following, we considered two variants of Model 2a, each assuming a different additional process that would account for the increased RT time, and tested whether these model variants also improve predictions.

**Fig 9:**
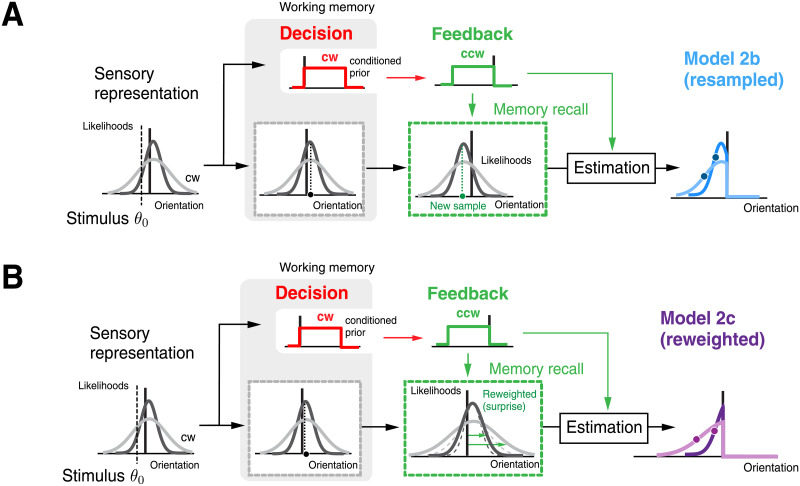
Hypothesis 2—Potential effects of receiving negative feedback on memory recall and estimation. (A) Model 2b (resampled) is identical to Model 2a (flip decision) except that it assumes sensory measurements are resampled from working memory until a new sample lies on the correct side of the reference orientation (i.e. is consistent with feedback). A new likelihood function based on this sample is then combined with the updated categorical prior to compute a Bayesian estimate. (B) Model 2c (reweighted) assumes that the level of surprise the observer experiences about the feedback will cause a reweighting of the sensory evidence: The larger the surprise of being wrong the more the observer reduces their belief in the sensory evidence in the estimation process. We modeled this belief change process by scaling the likelihood width proportionally to the level of surprise, measured as the divergence in beliefs between expected and resulting decision outcome. See Methods for details.

**Fig 10:**
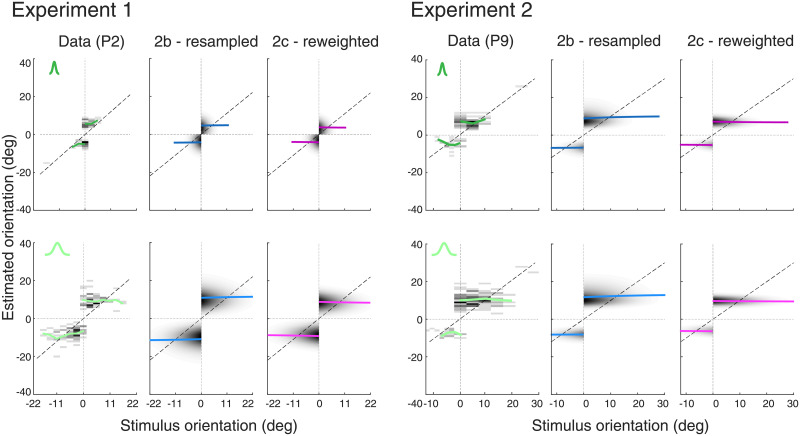
Predictions of Models 2b (resampled) and 2c (reweighted). Both model variants provide good predictions of the estimate distributions in incorrect trials of subject P2 (Experiment 1) and P9 (Experiment 2), respectively. Compared to Model 2a, the predicted mean estimates (solid lines) are larger and more accurately reflect the data. Note that Model 2b predicts a weak positive dependence between the mean estimate and true stimulus orientation, which may reflect a tendency in the data although with no significance (see S2 and S4 Figs for all other subjects). Model 2c predicts a weak negative dependence.

New Model 2b (resampling) assumes that memory recall is also based on a random sampling process (like Model 2a) but is constrained to only accept samples that are consistent with the top-level categorical interpretation. This represents a slightly modified self-consistent observer model where the categorical judgment not only acts as a top-down prior but also as a constraint on memory recall (see Fig 9A). The modification does not lead to any noticeable difference in quality of fit and fit model parameters when fitting data of correct trials in both experiments (see S7 Fig). For incorrect trials, however, resampling leads to estimate distributions that are, on average, more repulsed from the reference orientation than the predictions for Model 2a because now the peak, and not only the tail, of the likelihood function is on the correct side of the reference. This mends the main shortcoming of Model 2a. Fig 10 shows the predicted distributions together with the data for both Experiment 1 (subject P2) and 2 (subject P9), respectively.

New Model 2c, the second variant, makes the additional assumption that negative feedback reduces an observer’s belief in the stored sensory evidence. In particular, we assume that the more surprised the observer is about receiving negative feedback the less they trust their sensory evidence (Fig 9B). We implement this reweighting of the sensory evidence by scaling the width of the likelihood function with a measure of surprise that is based on the divergence in beliefs between expected and resulting decision outcome [26]. The reweighting does not occur in correct trials because in correct trials there is no surprise: Feedback simply confirms that the most likely decision outcome is the one that the observer chose. For incorrect trials, however, the level of surprise depends on the probability of making an incorrect decision based on the measurement *m*. Intuitively, one might therefore expect that the surprise is larger for stimulus orientations further away from the reference. This is true if we were interested in characterizing the surprise of the experimenter who knows the true stimulus value *θ*. Here, however, we are concerned about the level of surprise of the observer. The observer has no access to ground-truth *θ* but only to the stored sensory information (i.e. the likelihood function) and the negative feedback. The surprise for the observer thus only depends on how far *m* is from the decision boundary and the overall level of sensory uncertainty. As a result, the predicted estimate distributions for incorrect trials are generally more repulsed from the reference because all incorrect trials are associated with a certain level of surprise that reality did not match the most likely categorical judgment and thus with wider likelihood functions (Fig 10). For small values of *θ* there is a higher probability that the sensory measurements *m* is further away from the decision boundary than for large *θ*. Thus, somewhat counter-intuitive, the observer’s surprise for receiving negative feedback is generally larger in those incorrect trials where *θ* was close to the reference, culminating in more repulsed estimates for those stimulus orientations.

**Fig 11:**
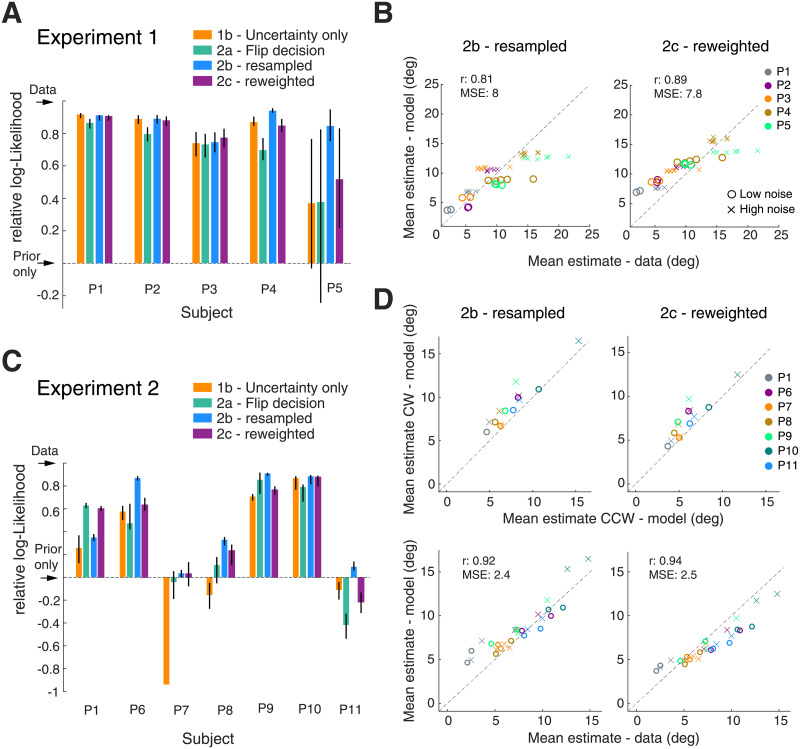
Performance of Models 2b (resampled) and 2c (reweighted). (A) Normalized log-likelihood comparison for Experiment 1. All models perform well compared to Model 1a (prior only), confirming that subjects maintain and reuse information about stimulus uncertainty on a per trial basis. Models 2b and 2c, however, perform consistently better than Model 2a. (B) Both models better predict subjects’ mean estimates than Model 1b (MSE and correlation; see Fig 5C). Predictions of Model 2c show a similar deviation from the data as the predictions of Model 2a. (C) Normalized log-likelihood comparison for Experiment 2. Model 2b consistently, but not always significantly, best predicts the data except for P1 (non-naïve). (D) In contrast to Model 1b, Models 2b and 2c both correctly predict mean estimates higher on the cw than the ccw side (see Fig 8B). Correlation and MSE measures indicate little difference between Model 2b and 2c; their predictions are better than those of Model 1b and 2a (see Fig 8C). All model predictions are parameter free and based on the parameter values of the fit self-consistent observer model to the data in correct trials. Errorbars indicate 95% confidence intervals computed from 200 bootstrapped samples.

Fig 11 quantifies the effect of resampling and reweighting sensory information on prediction accuracy for all subjects in both experiments. In terms of model likelihood, both variants are slightly but consistently better than the original Model 2a in predicting the incorrect trial data in Experiment 1 (Fig 11A; significant for 3 out of 5 subjects). A comparison of the predicted with the actual mean estimates of subjects confirms this assessment, showing that Models 2b and 2c deliver better predictions (in terms of MSE and correlation) than Model 2a and also Model 1b (see Figs 5C and 11C). Although there is little to separate the two model variants in terms of these quantitative metrics, the predicted mean estimates of Model 2c characteristically deviate from the data: Similar to the predictions of Model 2a, the predictions for subjects with small mean estimates are too high while they are too low for subjects with large mean estimates. Thus, the improved predictions of Model 2c are mainly due to overall larger estimates and not due to a correction of how the predictions scale for different mean estimates. Performing the same analysis for the data of Experiment 2 leads to a similar assessment (Fig 11C and 11D). For most subjects, Models 2b and 2c outperform Model 1b and 2a in terms of model likelihood but certainly also in prediction quality of subjects’ mean estimates (MSE and correlation). Again, the predictions of Model 2c (reweighting) exhibit the same systematic scale offset across both experiments as those of Model 2a, while Model 2b (resampled) does not show such consistent deviation (Fig 11B and 11D).

We conclude that subjects’ behavior in our experiments is well explained by a self-consistent Bayesian observer model where sensory information is fully and independently maintained in working memory even after the observer performs a categorical judgment about the stimulus, and can be flexibly recombined with categorical prior information on a per trial basis. A model variant that further assumes that recall of the sensory information is constrained to be consistent with the (negative) feedback best (and in fine detail) predicts the behavioral data across all individual subjects.

## Discussion

Previous studies have demonstrated how a high-level, categorical interpretation of a stimulus biases a subject’s subsequent recall of a low-level sensory feature of the stimulus. In this paper, we tested whether these biases directly reflect changes to the working memory representation of the stimulus or, alternatively, are the result of a perceptual inference process downstream from working memory. We ran two human psychophysical experiments that allowed us to measure the impact of subjects’ categorical assessments about the average orientation of a visual stimulus on their subsequent recall of the stimulus’ orientation under different noise conditions and stimulus distributions. By providing feedback about their categorical judgments we were able to probe subjects’ memory recall in trials in which they were incorrect in their initial judgment and had to switch their high-level interpretation of the stimulus accordingly. Using a strong cross-validated model comparison, we found no evidence that subjects’ initial (incorrect) judgments lastingly modified working memory representation of the stimulus. Rather, we found that subjects were able to flexibly recombine the original sensory information in working memory with the updated categorical interpretation (i.e. the feedback) and its associated prior distribution in their perceptual recall process. Data from both experiments were remarkably well predicted by a modified self-consistent observer model that conditions recall and inference to be consistent with the provided feedback.

It is possible that our findings may be limited to the specific context of the task in our experiments: Subjects knew they would receive reliable feedback in every trial and thus may have maintained the full sensory information only because of the prospect of later combining it with the feedback information. While we cannot rule out this possibility based on current empirical evidence, it seems rather unlikely given that it is the natural *modus operandi* of human perception and cognition to continually update beliefs whenever new information is available. New information can come any time (or not), can be both, affirmative or contradictory, and can also arrive at different levels of description (e.g. information at the category level while maintaining beliefs at the feature level). Thus under natural conditions it generally seems sensible to independently maintain beliefs in working memory, at any level, and in a form that allows the system to continually update and flexibly combine and recombine them as needed. Future empirical work will be required to ultimately validate this assumption.

Our results have implications that generalize beyond the specifics of our experiments. Firstly, estimation biases were substantially different for high- compared to low-noise stimulus conditions. Given that noise conditions were randomly changed on each trial, this suggests that working memory representations of stimulus features include the representation of trial-by-trial sensory uncertainty. This confirms conclusions from previous studies [10, 22, 25, 27], but furthermore also demonstrates that subjects can flexibly reuse this uncertainty information within a single trial.

Secondly, the ability to flexibly maintain and recombine probabilistic information is shared with other models suggesting that humans can efficiently reuse parts of previous inference processes in subsequent inference steps [28, 29]. However, in contrast to our model these “amortized inference” models assume recombinations at the level of posterior distributions, which cannot account for our current experimental results nor those of previous studies [7, 8, 10].

The demonstrated flexibility of human subjects to recombine information at different levels of the representational hierarchy has also implications for our general understanding of visual working memory. While previous results suggest that working memory representations are tightly linked across different levels of a hierarchical stimulus representations (see review [30]) our findings show that these links must remain sufficiently flexible at the time of recall.

Thirdly, the model that best predicted subjects’ estimates in incorrect trials assumes that memory recall involves a feedback-consistent resampling process. Interestingly, such resampling is conceptually similar with ideas of retrospective shifts in attentional focus during memory recall [31]. Stimuli in our experiments consisted of an array of oriented line segments and thus their working memory representations likely reflect distributions rather than only the average orientation that we assumed for reasons of simplicity. Feedback can act as a retrospective attentional shift, boosting the accuracy of memory representations of those lines that are consistent with feedback, which then results in a bias in the recalled average representation towards the side consistent with feedback. Future work is needed to more thoroughly investigate the similarities between retrospective attention and the proposed resampling process within the context of Bayesian observer models.

Resampling may not be limited to conditions where a subject is forced to change the categorical judgment but rather may reflect a mechanism intrinsic to the type of hierarchical sequential inference considered in our study. If so, it would require a modification of the self-consistent observer model [10, 21]. However, both the original and the updated model provide near-identical fits to data from correct trials in our experiments (see S7–S9 Figs). It confirms that resampling has little effect on the estimate in trials in which subjects did not have to change their categorical interpretation of the stimulus. Thus we expect the updated model to provide an equally valid explanation of all the previous data sets [7, 8, 10] and therefore represent an improved implementation of the self-consistent observer model.

Finally, our results may provide new interpretations of some well-known bias phenomena in cognitive judgment and decision making. Hind-sight bias, for example, refers to subjects’ biases in recalling their initial assessment of an uncertain quantity upon receiving feedback [32–34]. After having received feedback about the correctness of their answer, subjects’ recall of their initial answer was significantly biased towards being more consistent with the feedback. This is generally consistent with our results. However, our results suggest that these biases are due to a conditioned inference process during memory recall rather than a direct, feedback-induced modification of memory representations [35]. As such, the important implication is that hind-sight bias may be malleable or can even be eliminated by appropriate, measured feedback.

We end by proposing the following hypothesis: Visual working memory reflects an accurate reverberation of the original visual stimulus that, however, is flexibly interpreted at the time of recall. Interpretation is the result of a hierarchical inference process that allows to potentially incorporate new knowledge (e.g. provided by feedback). As a result, the same memorized sensory information can be interpreted multiple times in different ways if necessary. Crucial, however, is that the interpretation is self-consistent such that a commitment at one level conditions inference at the rest of the hierarchy. Working memory recall is an active, reversible, but self-consistent inference process.

## Methods

### Ethics statement

The experiments were approved by the Institutional Review Board of the University of Pennsylvania under protocol #819634. All human subjects provided written informed consent.

### Experimental procedure

Eleven subjects (3 males, 8 females; subject P1 non-naïve) with normal or corrected-to-normal vision participated in the experiments.

*General procedure*: Subjects sat in a darkened room. Stimuli were presented on a special purpose computer monitor (VIEWPixx3D, refresh rate of 120 Hz and resolution of 1920 x 1080 pixels) at a distance of 83.5 cm (Experiment 1) or 91 cm (Experiment 2) enforced via a chin rest. Screen background luminance was 40 cd/m^2^ and mean stimulus luminance was 49 cd/m^2^. Both experiments and data collection were programmed in Matlab (Mathworks, Inc.) using the MGL (http://justingardner.net/mgl) and the Psychophysics Toolbox [36], and were run on an Apple Mac Pro computer with Quad-Core Intel Xeon 2.93 GHz and 32GB of RAM. Before each main experiment, subjects were extensively trained to get familiar with the task and to learn the stimulus distribution. After training, each subject either completed 2100 trials in 3–5 sessions for Experiment 1 or completed 1820 trials in 3–4 sessions for Experiment 2. Each session lasted approximately 50 minutes. In total, each stimulus condition was repeated 70 times and consisted of 15 (Experiment 1) or 13 (Experiment 2) stimulus orientations and two noise levels. Subjects used a gamepad (Sony PS4 Dualshock) to indicate their categorical judgments by pressing a trigger button (left for ‘ccw’, right for ‘cw’). To report the perceived stimulus orientation, subjects adjusted a reference line (length: 5 deg) with the analog joystick of the gamepad and then pressed a button for confirmation. Subjects were instructed to fixate whenever the fixation dot was present.

*Experiment 1*: Five subjects (P1–5) participated in Experiment 1. At the beginning of each trial, subjects were presented with a fixation dot (diameter: 0.3 deg) and a reference orientation indicated by two black lines (length: 3 deg, distance from fixation: 3.5 deg). We randomly selected the reference orientation from a uniform distribution [0, 180] deg in each trial. After 1 s, an orientation stimulus was presented, consisting of an array of white line segments (length: 0.6 deg) that were arranged on two circles around at the fixation dot: the outer circle (diameter: 3.8 deg) had 16 line segments and the inner circle (diameter: 1.8 deg) had 8 line segments. Small random jitters (from -0.15 to 0.15 deg) were independently added to the x-y coordinates of each line segment. We randomly sampled the orientation of each line segment from a Gaussian distribution whose mean (stimulus orientation) ranges from -21 (ccw) to 21 (cw) deg in steps of 3 deg relative to the reference orientation with standard deviations (stimulus noise) 3 and 18 deg. As a result, the sample mean of the array in every one trial was not necessarily identical with the stimulus orientation *θ* (generative mean). Stimuli were presented for 500 ms. Subjects were instructed to indicate whether the average array orientation was cw or ccw of the reference orientation. Auditory feedback (100% valid) was provided 500 ms after subjects’ response to indicate whether the categorical judgment about the stimulus orientation was correct. Subjects then went on to report their perceived stimulus orientation. They were instructed that the feedback was always valid and should be taken into account when performing the estimation task. If subjects took longer than 4 s to provide the categorical judgment then the current trial was skipped and was moved to the end of the trial queue. At the end of each trial, a blank screen (mean luminance) was displayed with a duration randomly chosen from 300 ms to 600 ms.

*Experiment 2*: Seven subjects (P1, P6–11) participated in Experiment 2. The experimental design was identical to Experiment 1 except for the following differences: Stimulus range was [-12, 0] deg in steps of 2 deg for the ccw side and [0, 30] deg in steps of 5 deg for the cw side relative to the reference orientation. We used different step sizes for the two sides to keep the total number of stimulus orientations on both sides the same. Also, subjects were reminded of the (asymmetric) stimulus range at the beginning of each trial for both training and main experiments (see Fig 6A). Standard deviation of the low-noise condition was 6 deg compared to 3 deg in Experiment 1.

*Training—simple motor task*: Subjects were first trained to perform the estimation task. In each training trial, subjects first fixated their gaze on the fixation dot. For subjects participating in Experiment 2, we also displayed the decision boundary (like in the main experiment) and a gray arc indicating the asymmetric stimulus range during fixation. After fixation only the fixation dot remained and a white line (stimulus) appeared for 500 ms. Subjects then had to reproduce the white line by adjusting a line cursor with the analog joystick. After confirming their response by pressing a button, the original stimulus was displayed in green on top of subjects’ estimates. The decision boundary was uniformly sampled around the circle and the stimulus orientation was uniformly sampled in the same range as in Experiment 1 and 2, respectively. Subjects completed 450 trials (Experiment 1) or 325 trials (Experiment 2). We computed the standard deviation of subjects’ estimates and used that as the individual motor noise parameter in the model fits and predictions performed in our analysis.

*Training—estimation task*: After the first training session, a second training session was performed to accustom subjects with the stimulus estimation task. Trials were identical to the motor training except that we used the array stimulus in the main experiment instead of the single line stimulus. Subjects completed 100 trials (Experiment 1) or 150 trials (Experiment 2) for this training.

*Training—decision task*: The third training session was aimed at training the categorical judgment task. Stimuli were identical to estimation training but subjects were tasked to perform a categorical judgment (cw/ccw). Feedback was provided like in the main experiments. Subjects completed 900 trials (Experiment 1) or 200 trials (Experiment 2).

### Data

The trial data was separated into two categories, correct and incorrect trials, based on whether subjects’ initial categorical judgments were correct or not. In trials where the stimulus orientation was identical to the reference orientation, the correct stimulus category was randomly assigned a priori. For a very small fraction of trials (0.77% for Experiment 1 and 0.46% for Experiment 2), subjects’ estimates were inconsistent with either their correct categorical judgment or the feedback (incorrect trials). We have previously demonstrated that these inconsistent trials are fully explained by attentional lapses and/or motor noise [10]. In the current study, we excluded data from these very few trials for reasons of simplicity.

### Self-consistent Bayesian observer model

The key assumption of the model is that after making a categorical judgment, the observer fully conditions the subsequent estimation process on that subjective decision. The model is described in detail in [10], but for the reader’s sake we provide a summary description below.

#### Decision task

A key assumption of the model is that the observer considers the stimulus in the context of a hierarchical generative model. Let *C* = {‘cw’, ‘ccw’} be the category variable indicating whether the stimulus orientation is cw or ccw relative to a reference line and *θ* be the low-level stimulus feature representing the mean orientation of the line array. Furthermore, the model assumes that on each trial the observer forms a noisy sensory measurement *m* representing the stimulus orientation *θ*. Also, the observer knows the sensory uncertainty associated with that particular measurement *m* represented as a likelihood function *p*(*m*|*θ*). Assuming that the observer follows an optimal decision process, the likelihood function over *C* is given as

$$\begin{array}{c} p(m|C)=\int p(m|\theta )p(\theta |C)d\theta \;. \end{array}$$ (1)

where *p*(*θ*|*C*) represents the observer’s prior expectation about the stimulus orientation on the two sides of the decision boundary. The observer then computes the posterior over the decision variable by combining the likelihood and the prior *p*(*C*):

$$\begin{array}{c} p\left(C|m\right)=\frac{p(m|C)p(C)}{p(m)} \end{array}$$ (2)

Given a symmetric loss function, the observer chooses the category with the higher posterior, thus

$$\begin{array}{c} \hat{C}\left(m\right)={\text{argmax}}_{C\in \{‘\text{cw}’,‘\text{ccw}’\}}p\left(C|m\right)\;. \end{array}$$ (3)

Note, that we assume the parameters of the likelihood function and the prior to be “subjective”, i.e., determined by the particular noise in the observer’s sensory process and their probably imperfect knowledge of the stimulus statistics. In that sense, “optimal” refers to the observer’s best knowledge and not to some sort of absolute ground-truth [37]. We can obtain the model prediction of the psychometric function by marginalizing over the sensory measurement distribution:

$$\begin{array}{c} p\left(\hat{C}|\theta \right)=\int p\left(\hat{C}|m\right)p\left(m|\theta \right)dm\;. \end{array}$$ (4)

#### Estimation task

After making the categorical decision, the observer’s task is to estimate the stimulus orientation. We assume that the sensory measurement *m* has been held in visual working memory since the stimulus disappeared and that at the time of estimation, *m* is recalled from memory with some additional uncertainty due to memory degradation. We refer to the recalled sample as *m*_*m*_ and assume that memory degradation is described by the distribution *p*(*m*_*m*_|*m*). The distribution of memory samples for a given stimulus orientation is thus given as

$$\begin{array}{c} p\left(m_{m}|\theta \right)=\int p\left(m_{m}|m\right)p\left(m|\theta \right)dm\;. \end{array}$$ (5)

A fundamental aspect of the model is that the observer considers their own subjective categorical judgment as if it was factual. As a result, the preceding category judgment determines the prior distribution in the estimation task referred to as the conditioned prior $p(\theta |\hat{C})$. Thus the resulting estimate is determined by computing the posterior based on the likelihood function (Eq (5)) and this conditioned prior,

$$\begin{array}{c} p\left(\theta |m_{m},\hat{C}\left(m\right)\right)\propto p\left(m_{m}|\theta \right)p\left(\theta |\hat{C}\left(m\right)\right)\;, \end{array}$$ (6)

and then applying a particular loss function. Unless indicated otherwise, we assume an *L*_2_ norm loss, thus

$$\begin{array}{c} \hat{\theta }\left(m_{m},\hat{C}\left(m\right)\right)=\int \;\theta \;p\left(\theta |m_{m},\hat{C}\left(m\right)\right)d\theta \;. \end{array}$$ (7)

### Model predictions for incorrect trials

In testing their ability to correctly predict subjects’ estimates in those trials where their categorical judgment was incorrect, we can now formulate and compare different models that address the question whether categorical judgments lead to an update of working memory representations or not. Note that for correct trials, behavior is well described by the self-consistent observer model outlined above. For incorrect trials we considered the following models based on our two alternative hypotheses (Fig 1):

#### Hypothesis 1: Categorical judgments update memory representations

We assume that the categorical judgment $\hat{C}$ modifies sensory information in working memory by updating the likelihood function to be consistent with the posterior. After receiving negative feedback, we considered the two following models for how the observer potentially would form a stimulus estimate $\hat{\theta }$ in this situation (Fig 2).

*Model 1a (prior only)*: Updating the likelihood function based on $\hat{C}$ can be written as

$$\begin{array}{c} p_{new}\left(m_{m}|\theta \right)=p\left(m_{m}|\theta \right)p\left(\theta |\hat{C}\right)\;, \end{array}$$ (8)

which erases sensory information that is inconsistent with the decision (i.e., the likelihood function is set to zero/uniform for that stimulus range). Thus, if feedback indicates that $\hat{C}$ was incorrect, the posterior for those incorrect trials is equivalent to the prior distribution according to the feedback corrected judgment ${\hat{C}}_{f}$, thus

$$\begin{array}{c} p\left(\theta |m_{m},{\hat{C}}_{f}\right)\propto p\left(\theta |{\hat{C}}_{f}\right) \end{array}$$ (9)

Assuming an *L*_2_ loss function, the optimal estimate is represented by the mean of the posterior, i.e. the mean of the prior, thus

$$\begin{array}{c} {\hat{\theta }}_{1a}\left(m_{m},{\hat{C}}_{f}\right)=\int \;\theta \;p\left(\theta |m_{m},{\hat{C}}_{f}\right)d\theta =\int \;\theta \;p\left(\theta |{\hat{C}}_{f}\right)d\theta \;. \end{array}$$ (10)

As a result, the estimates are independent of stimulus uncertainty and the variance is only determined by the motor noise of individual subjects (see S11 Fig).

*Model 1b (uncertainty only)*: Although the observer has no longer access to specific information about stimulus orientation on the correct side of the reference, it still has a sense of the overall level of sensory uncertainty in a trial. The purpose of this model is to test, in conjunction with Model 1a, the degree to which each source of information, sensory uncertainty and the prior distribution, is reflected in subjects’ estimates after receiving negative feedback.

Specifically, Model 1b is a heuristic that assumes that the observer picks an estimate that is *σ*_*s*_ away from the reference orientation. Thus model estimates only depend on stimulus uncertainty and are independent of stimulus value *θ* and stimulus prior. The variance of the estimate distributions $p(\hat{\theta }|\theta )$ only depends on the observer’s motor noise. This heuristic constitutes a crude approximation of subjects’ behavior in correct trials where larger stimulus noise results in larger biases away from the reference orientation.

#### Hypothesis 2: Sensory representation in working memory is preserved

Alternatively, we considered that sensory information is unaltered in working memory. Thus the information stored is equivalent to the original measurement *m* and some representation of uncertainty about that value (e.g. the likelihood function *p*(*m*|*θ*)). The general assumption is that the observer simply uses the category judgment ${\hat{C}}_{f}$ provided by feedback rather than the initial, incorrect judgment $\hat{C}$ when performing the estimate. However, we consider two different versions of how working memory is recalled for this inference process.

*Model 2a (flip decision)*: Memory recall is as described above leading to a likelihood function (Eq (5)) that represents additional uncertainty due to memory degradation. Inference is according to the self-consistent observer model conditioned, however, on the feedback categorical judgment ${\hat{C}}_{f}$, thus

$$\begin{array}{c} p\left(\theta |m_{m},{\hat{C}}_{f}\right)\propto p\left(m_{m}|\theta \right)p\left(\theta |{\hat{C}}_{f}\right)\;. \end{array}$$ (11)

The estimate $\hat{\theta }\left(m_{m},{\hat{C}}_{f}\right)$ is then the mean of the posterior under *L*_2_ loss function.

*Model 2b (flip decision, resampled)*: This model is identical to Model 2a except that memory recall does not follow the distribution (5) but involves a conditioned sampling process $p(m_{m}|m,{\hat{C}}_{f})$ such that it only accepts memory recalls *m*_*m*_ that are consistent with ${\hat{C}}_{f}$. We assume that samples are repeatedly drawn until the sample lies on the correct side of the decision boundary.

*Model 2c (flip decision, reweighted)*: This model is identical to Model 2a except that the likelihood width is scaled by a factor that is proportional to the level of surprise of the observer about receiving negative feedback on the categorical judgment.

We applied a Bayesian definition of surprise [26], defined as the KL divergence between the posterior distribution over the chosen decision $p(\hat{C}|m)$ and the opposite choice $p(\neg \hat{C}|m)$:

$$\begin{array}{c} KL(p\left(\hat{C}|m\right)||p\left(\neg \hat{C}|m\right))=\sum_{\hat{C}}p\left(\hat{C}|m\right)\text{log}\frac{p(\hat{C}|m)}{p(\neg \hat{C}|m)}\;. \end{array}$$ (12)

Note that this formulates a surprise signal from the observer’s perspective as it is defined in terms of the sensory measurement *m*. The likelihood for computing the estimate is then given by the reweighted sensory uncertainty $\sigma_{s}^{\prime }$ and the memory noise *σ*_*m*_ where $\sigma_{s}^{\prime }=\sqrt{1+KL}\;\sigma_{s}$.

For all models under Hypothesis 2, the predictive estimate distribution are computed by marginalizing over the distribution of memory samples (or the resamples)

$$\begin{array}{c} p\left(\hat{\theta }|\theta ,\hat{C}\right)=\int p\left(\hat{\theta }|m_{m},\hat{C}\right)p\left(m_{m}|\theta \right)dm_{m}\;, \end{array}$$ (13)

and then again marginalizing over the distribution of decision outcome (the psychometric function), thus

$$\begin{array}{c} p\left(\hat{\theta }|\theta \right)=\sum_{\hat{C}}p\left(\hat{\theta }|\theta ,\hat{C}\right)p\left(\hat{C}|\theta \right)\;. \end{array}$$ (14)

Finally, we convolve the corresponding predictive distributions with a motor noise kernel that was independently determined for each subject based on their individual motor training data (see above).

#### Model specification

Note, model predictions were entirely parameter-free. Model parameters were based on fits of the self-consistent model on the correct trial data. The model contains the following parameters:

- The category prior *p*(*C*) is fixed to be 0.5 for Experiment 1 because of symmetry reasons. However, we leave this as a free parameter for Experiment 2 because the asymmetric stimulus range may induce a non-uniform category prior (even though objectively *p*(*C* = ‘cw’) = *p*(*C* = ‘ccw’)).
- The categorical prior distributions over stimulus orientation *p*(*θ*|*C*) are either symmetric (Experiment 1) or asymmetric (Experiment 2) around the reference orientation. More specifically, *p*(*θ*|*C*) is assumed to be constant up to a certain orientation away from the reference on each side, and then monotonically decreases to zero with a cosine roll-off. So the prior distribution is characterized by two parameters: the prior width *α*, which is the size of the uniform range and a smoothing factor *β*, which indicates how smooth the roll-off is. For the purpose of fitting and predicting the data, the role of *β* is relatively small compared to the prior width *α*. Therefore, we only show the prior width in our plots of model parameters.
- The measurement distribution *p*(*m*|*θ*) is assumed to be Gaussian centered on the stimulus orientation *θ* and with standard deviation *σ*_*s*_ proportional to the sensory uncertainty. We consider *σ*_*s*_ a free parameter for each subject and noise level that represents the total sensory uncertainty representing the combination of stimulus noise (variance in the line-array of the stimulus display) and some internal, neural noise. We assume that *σ*_*s*_ only depends on the stimulus noise and is fixed across all other experimental conditions.
- We assume working memory to be noisy such that the recalled sensory measurement *m*_*m*_ represents a noisy sample of the original measurement *m*. Noise is modeled according to a Gaussian *p*(*m*_*m*_|*m*) centered on the original sensory measurement *m* with standard deviation *σ*_*m*_. The latter is assumed to be a free parameter for each subject but fixed for each subject across all experimental conditions.
- For Model 2b, the resampling distribution *p*(*m*_*m*_|*m*) is centered on the memory sample *m* and has the standard deviation $\sqrt{\sigma_{s}^{2}+\sigma_{m}^{2}}$. The resampling distribution has the combined variance because the observer takes into account the fact that for higher sensory noise, the original stimulus orientation is more likely to be farther away on the correct side. We assume resampling to be an active process in which the observer attempts to reconstruct the sensory information on the correct side.
- The motor noise is modeled as a Gaussian with standard deviation *σ*_0_ that was determined from the motor noise training data for each individual subject (see S11 Fig).

#### Omniscient model

We computed an upper bound for model likelihood comparisons using the prediction of an ‘omniscient’ model (Figs 5B, 11B and 11C). The bound can be thought of as the “data explaining themselves”, that is, a probabilistic model whose conditional probabilities $p(\hat{\theta }|\theta )$ are constructed from the empirical distribution of subjects’ estimates. We approximated the empirical probability distributions by binning the data into equally sized bins (width of 0.5 deg) and determined the empirical probabilities for each bin by computing the fraction of data points within.

Note that for the model comparisons, we performed a similar discrete approximation for the model probabilities using the same bin center and bin width. However, instead of the fraction of data within a bin, we integrated the probability density distribution over the bin. We then computed the model likelihoods based on these discrete conditional probabilities.

#### Model fit and comparison

We performed a joint fit to all trials of the decision task and correct trials of the estimation task by maximizing the likelihood of model parameters given the data:

$$\begin{array}{c} p\left(D|\rho \right)=\prod_{i=1}^{n}P\left(D_{i}|\rho \right)=\prod_{i=1}^{n}P\left({\hat{C}}_{i}|\rho \right)\;\times \prod_{i|\hat{C_{i}}\;\text{correct}}^{n}p\left({\hat{\theta }}_{i}|\rho \right)\;, \end{array}$$ (15)

where *D* is the data, *ρ* is the parameters of the model, ${\hat{C}}_{i}$ is subjects’ decision, ${\hat{\theta }}_{i}$ is subjects’ orientation estimate, *i* is the trial index and *n* is the total number of trials. We used Nelder-Mead simplex optimization to minimize the negative log likelihood −*log*(*p*(*D*|*ρ*)). We ran the optimization routine thirty times starting at randomized initial parameter values to find the best parameter set.

## Supporting information

S1 Fig Experiment 1—Data and model fits of individual subjects (correct trials). (EPS)

S2 Fig Experiment 1—Data of individual subjects (incorrect trials). (EPS)

S3 Fig Experiment 2—Data and model fits of individual subjects (correct trials). (EPS)

S4 Fig Experiment 2—Data of individual subjects (incorrect trials). (EPS)

S5 Fig Experiment 1—Correlations between mean estimates and fit model parameters. The strong correlation between subjects’ mean estimates and their prior widths and sensory noise levels indicates that subjects’ estimates in incorrect trials are the result of an inference process that combines sensory information with prior expectations.
(EPS)

S6 Fig Experiment 2—Correlations between mean estimates and fit model parameters. The strong correlation between subjects’ mean estimates and their prior widths and sensory noise levels indicates that subjects’ estimates in incorrect trials are the result of an inference process that combines sensory information with prior expectations.
(EPS)

S7 Fig Self-consistent observer model fits of correct trial data with resampling. (A and B) Fit model parameters with resampling for data from Experiment 1 and 2 (Compare to Figs 5A and 8A, parameters without resampling). (C) Log-likelihood differences of the self-consistent observer model with and without conditioned resampling for each subject. For correct trials, fits of both versions of the model are essentially indistinguishable.
(EPS)

S8 Fig Model 2b fits to correct and predictions of incorrect trial data in Experiment 1 (subject P2). The top two rows show fits of the self-consistent observer model without resampling. Note, histograms for stimulus orientations far away from the reference orientation only contain very few incorrect trials. Thus they do not provide a test of the model predictions.
(EPS)

S9 Fig Model 2b fits to correct and predictions of incorrect trial data in Experiment 2 (subject P9). The top two rows show the fits of the self-consistent observer model without resampling. Note, histograms for stimulus orientations far away from the reference orientation only contain very few incorrect trials. Thus they do not provide a test of the model predictions.
(EPS)

S10 Fig Response time (RT) for subjects and the conditioned resampling model. (A) Subjects’ response times (RTs) in the estimation task (Experiment 2). RTs are measured as the time between confirming the decision and confirming the estimate with a button press on the game pad. RTs are substantially higher (difference: 430 ms) in incorrect trials compared to correct trials (averaged across all subjects). This indicates that negative feedback slows down subjects’ inference process, which could be caused by triggering additional processes such as resampling (Model 2b) or reweighting of sensory information (Model 2c). Also, RTs tend to be larger for higher noise condition. (B) Simulated increases in RTs of Model 2b. Increases in RTs are calculated as the relative number of samples necessary before a sample was acceptable (i.e. fell on the correct side of the reference). The simulated increases in RTs are qualitatively consistent with the data including the consistent (but not significant) trend that RTs are larger for high than for low stimulus noise conditions.
(EPS)

S11 Fig Motor noise of individual subjects extracted from the motor training session (see Methods for details). (EPS)
